# Immune Reactivity of a 20-mer Peptide Representing the Zika E Glycan Loop Involves the Antigenic Determinants E-152/156/158

**DOI:** 10.3390/v12111258

**Published:** 2020-11-05

**Authors:** Etienne Frumence, Juliano G. Haddad, Bénédicte Vanwalscappel, Jessica Andries, Jason Decotter, Wildriss Viranaicken, Gilles Gadea, Philippe Desprès

**Affiliations:** Unité Mixte Processus Infectieux en Milieu Insulaire Tropical, Plateforme Technologique CYROI, Université de La Réunion, INSERM U1187, CNRS UMR 9192, IRD UMR 249, 97491 Sainte-Clotilde, La Réunion, France; Etienne.frum@gmail.com (E.F.); juliano.haddad@univ-reunion.fr (J.G.H.); benedicte.vanwalscappel@univ-reunion.fr (B.V.); jessica.andries85@gmail.com (J.A.); decotterjason@gmail.com (J.D.); wildriss.viranaicken@univ-reunion.fr (W.V.); gilles.gadea@inserm.fr (G.G.)

**Keywords:** arbovirus, Zika virus, envelope protein, viral peptide, glycan loop region, peptide-based ELISA, immunogenicity, antigenic reactivity

## Abstract

Mosquito-borne Zika virus (ZIKV) causes a severe congenital syndrome and neurological disorders in humans. With the aim to develop a live-attenuated ZIKV strain, we generated a chimeric viral clone ZIKALIVax with African MR766-NIID strain as backbone and the envelope E protein of epidemic Brazilian BeH810915 strain. The MR766-NIID residues E-T152/I156/Y158 were introduced into BeH810915 E protein leading to a nonglycosylated ZIKALIVax. Recently, we reported that the residues E-152/156/158 that are part of ZIKV glycan loop (GL) region might have an impact on the availability of neutralizing antibody epitopes on ZIKV surface. In the present study, we evaluated the antigenic reactivity of a synthetic 20-mer peptide representing the ZIKALIVax GL region. The GL-related peptide was effective for the detection of GL-reactive antibody in mouse anti-ZIKALIVax immune serum. We showed that the residue E-158 influences the antigenic reactivity of GL-related peptide. The ZIKALIVax peptide was effective in generating mouse antibodies with reactivity against a recombinant E domain I that encompasses the GL region. The GL peptide-reactive antibodies revealed that antigenic reactivity of E-domain I may be impacted by both residues E-152 and E-156. In conclusion, we proposed a role for the residues E-152/156/158 as key antigenic determinants of ZIKV glycan loop region.

## 1. Introduction

Mosquito-borne ZIKV belonging to flavivirus genus of *Flaviviridae* family is the etiologic agent of Zika congenital syndrome and neurological disorders in humans [1,2,3,4,5]. ZIKV strains are mostly clustered into African and Asian lineages [6,7]. In the past decade, there has been unexpected expansion of the geographic distribution of ZIKV strains of Asian lineage and their rapid spread caused major epidemics in the South Pacific in 2013 and then South America including Brazil in 2015 [2,7,8]. Contemporary epidemics ZIKVs have been associated with birth defects as well as contaminations through various human body fluids [3,4,5,8].

Vaccination has been proposed as an efficient strategy to prevent ZIKV infection in humans [9,10,11]. It is now well established that elicitation of a protective antibody response is a critical step in the development of safe and efficient Zika vaccines [12,13,14,15]. The envelope E protein (504 aa) is responsible for virus entry into the host-cell and represents a major target for ZIKV neutralization [16,17,18,19,20,21,22,23,24,25,26,27]. The ZIKV E ectodomain (residues E-1 to E-406) is divided into three structural envelope domains: Domain I (EDI), Domain II (EDII), and Domain III (EDIII) [1,9,17,19,25]. As depicted in Figure 6, the EDI domain contains 132 residues distributed in three spaced segments: The N-terminal residues E-1 to E-52, the central residues E-132 to E-193, and the C-terminal residues E-280 to E-296 [17,25]. EDI encompasses a flexible glycan loop GL (residues E-145 to E-164) region, which may be post-translationally N-glycosylated at N154 [27,28,29]. The 20 amino acids that compose Zika GL might have an influence on the conformation of E, in particular, on the accessibility of EDII, which encompasses the fusion loop region [30,31,32,33,34]. An important role has been also proposed for GL in relation with antigenic properties of Zika E protein [24,25,26].

ZIKALIVax (also called ZIKBeHMR-2) is a chimeric viral clone with historical African strain MR766-NIID (Genbank access LC002520) as backbone and structural protein region from epidemic Brazilian viral strain BeH810915 (Genbank access KU365778) [35,36]. Similar to BeH810915, Asian-lineage ZIKV isolates associated with recent epidemics share the common sequon N154/D155/T156, located in the GL region where an N-glycan is attached to N154 [29,30]. Unlike what has been described with Asian-lineage ZIKV, the residue N154 of African-lineage MR766-NIID E protein lacks N-glycan due to residue Ile at the position E-156, leading to a loss of the sequon [32]. In view of the importance of N-glycosylation status in the virulence of flaviviruses including ZIKV, ZIKALIVax was designed as a nonglycosylated chimeric virus [36]. Consequently, the residues I152/T156/H158 present in BeH810915 E protein were replaced with the MR766-NIID residues T152/I156/K158 [36]. The residue Ile at position E-156 results in a loss of the unique N-glycosylation site into GL region leading to a nonglycosylated ZIKALIVax [36].

We reported that inoculation of live ZIKALIVax in adult BALB/c mice resulted in production of neutralizing anti-ZIKV E antibodies [36]. While ZIKALIVax induced anti-E antibodies that neutralize MR766-NIID with high titers by plaque reduction neutralization test and flow-cytometry neutralization test (FNT), the epidemic ZIKV strains of Asian lineage were weakly neutralized by anti-ZIKALIVax immune serum [36,37]. As BeH810915 and ZIKALIVax E proteins only differ by the amino-acid substitutions at positions E-152/156/158, we questioned whether these three residues, which are part of the GL region, influence the availability of neutralizing antibody epitopes on ZIKV surface (36). In the present study, we sought to evaluate the antigenic reactivity of a synthetic peptide representing the ZIKALIVax GL region. A recombinant E-Domain I (rEDI), which encompasses the GL region of ZIKALIVax, was also generated. Both GL-related peptide and rEDI were mutated to assess the impact of residues E-152/156/158 on immune reactivity of antibodies raised against the glycan loop region. Our results showed that residues E-152/156/158 are key determinants in antigenic reactivity of ZIKV GL region.

## 2. Results

### 2.1. Reactivity of a Synthetic Peptide Representing the ZIKALIVax Glycan Loop Region

**Figure 1:**
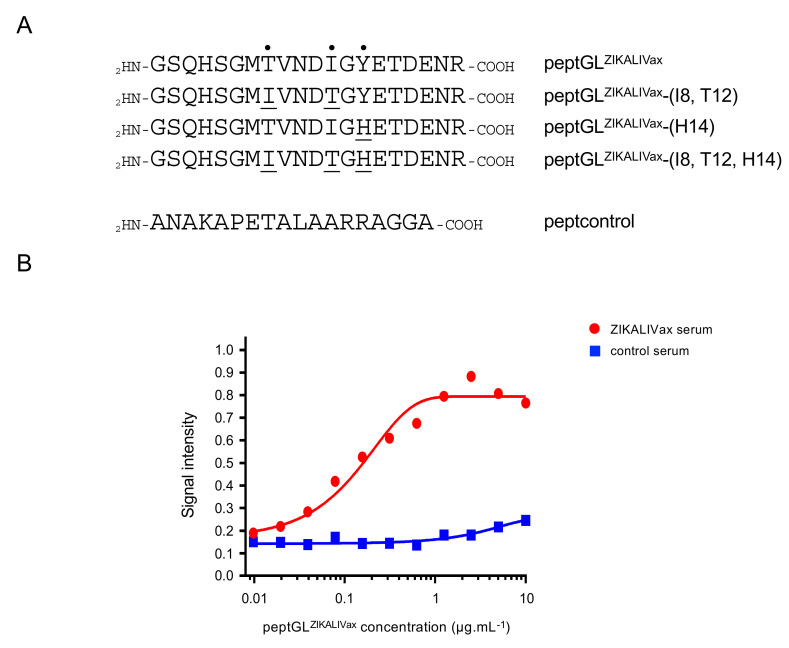
Reactivity of a peptide representing the ZIKALIVax glycan loop region through peptide-based ELISA. In (**A**), sequences of synthetic peptides representing the GL region from viral clone ZIKALIVax and mutants. The black points and underlined amino acids are positions that differ from peptGL^ZIKALIVax^. The irrelevant peptide peptcontrol is indicated. In (**B**), the optical density at 450 nm (O.D_450)_ or signal intensity of samples tested in a dose-response curve of peptGL^ZIKALIVax^ in relation to anti-ZIKALIVax immune serum through peptide-based ELISA. Plates were coated with increasing concentrations of peptGL^ZIKALIVax^ and incubated with a pooled anti-ZIKALIVax immune serum (ZIKALIVax serum) at dilution 1:50. A pool of serum of mice that received heat-inactivated ZIKALIVax [33] at dilution 1:50 served as a negative serum control (control serum).

We sought to evaluate the antigenic reactivity of a 20-mer synthetic peptide named peptGL^ZIKALIVax^ representing the GL region (residues E-245 to E-264) of chimeric viral clone ZIKALIVax (Figure 1A). To investigate the impact of residues E-152/156/158 on antigenic reactivity of peptGL^ZIKALIVax^, a mutant peptGL^ZIKALIVax^ peptide bearing the amino acid changes Thr to Ile at position 8 (residue 152 as numbered in BeH819015 E protein), Ile to Thr at position 12 (residue E-156), and His to Tyr at position 14 (residue E-158) was generated (Figure 1A). It is of note that peptide mutant peptGL^ZIKALIVax^-(I8, T12, H14) has complete similarity with BeH819015 GL region. An Ala-rich peptide of 18 amino-acid residues (hereafter titled peptcontrol), which lacks potential B-cell epitope, served as a negative peptide control.

A pool of sera of mice that received live ZIKALIVax has ability to bind peptGL^ZIKALIVax^ by peptide-based ELISA (Figure 1B). As a negative control, no binding was observed with a pooled serum of mice that received heat-inactivated ZIKALIVax. A dose-response curve showed that peptide detection limit was about 0.1 µg mL^−1^, whereas the plateau was reached at 1 µg mL^−1^ (Figure 1B). Thus, a peptide-based ELISA using peptGL^ZIKALIVax^ is suitable for detection of GL-reactive antibodies in mouse anti-ZIKALIVax immune sera. A concentration of 3 µg mL^−1^ (300 ng per well of a 96-well plate) of peptGL^ZIKALIVax^ was used for peptide-based ELISA in the following experiments.

**Figure 2:**
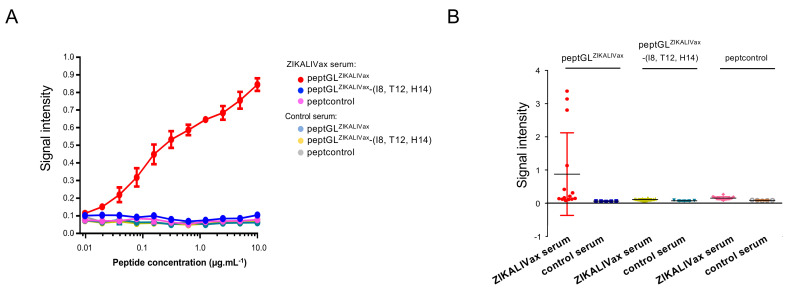
Immunoreactivity of pept^GLZIKALIVax^. The O.D_450_ or signal intensity of samples in dose-response curves of peptGL^ZIKALIVax^ and mutant peptide peptGL^ZIKALIVax^-(I8, T12, H14) in relation to anti-ZIKALIVax immune serum (ZIKALIVax serum) at dilution 1:200 measured through peptide-based ELISA. Serum of mice that received heat-inactivated ZIKALIVax at dilution 1:200 served as a control serum. Peptcontrol peptide was used as a negative peptide control. In (**A**), plates were coated with increasing concentrations of peptides and incubated with a pooled ZIKALIVax immune serum. In (**B**), peptides at concentration of 3 µg mL^−1^ were incubated with 14 individual anti-ZIKALIVax immune sera.

We next examined the antigenic reactivity of anti-ZIKALIVax immune serum against mutant peptGL^ZIKALIVax^-(I8, T12, H14) (Figure 2A). Heat-inactivated ZIKALIVax immune serum served as negative serum control, whereas peptGL^ZIKALIVax^ and peptcontrol were used as positive and negative peptide controls, respectively. There was no antigenic reactivity of anti-ZIKALIVax immune serum against mutant peptGL^ZIKALIVax^-(I8, T12, H14) regardless of the peptide concentration tested (Figure 2A). Thus, anti-ZIKALIVax immune serum had peptide-reactive antibodies raised against peptGL^ZIKALIVax^ but not a mutant peptide representing the BeH819015 GL region.

We examined the antigenic reactivity of 14 individual anti-ZIKALIVax immune sera against peptGL^ZIKALIVax^ and mutant peptGL^ZIKALIVax^-(I8, T12, H14) by peptide-based ELISA (Figure 2B). The GL-reactive antibodies were readily detected for one-third of serum samples, whereas no recognition was observed with mutant peptide regardless of individual serum tested. Thus, individual variation in antibody response to peptGL^ZIKALIVax^ can occur in inbred laboratory mice inoculated with live ZIKALIVax.

**Figure 3:**
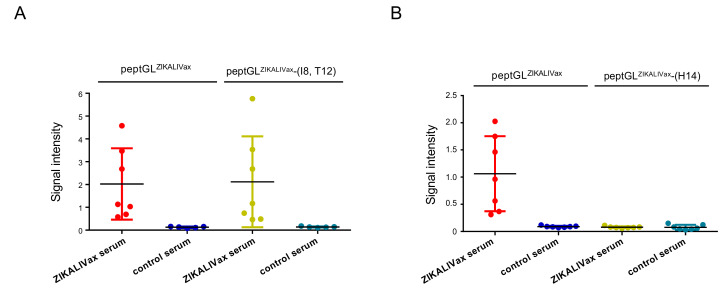
Immunoreactivity of peptGL^ZIKALIVax^ mutants with anti-ZIKALIVax immune serum. The O.D_450_ or signal intensity of individual anti-ZIKALIVax immune sera in relation to peptGL^ZIKALIVax^ and its mutants at positions 8 and 12 (**A**) or 14 (**B**) measured through peptide-based ELISA. Peptides at concentration of 3 µg mL^−1^ were incubated with individual anti-ZIKALIVax immune sera (*n* = 7) (ZIKALIVax serum) showing the higher reactivity with peptGL^ZIKALIVax^ at dilution 1:200 (Figure 2B). Serum of mice (*n* = 7) that received heat-inactivated ZIKALIVax at dilution 1:200 served as a control serum. Each closed circle represents an individual mouse.

As shown in Figure 1A, the peptide peptGL^ZIKALIVax^ is composed of amino-acids T8 and I12 but I8 and T12 in mutant peptGL^ZIKALIVax^-(I8, T12, H14). We asked whether amino-acid permutation (Thr, Ile) at positions 8 and 12 might play a role in antigenic reactivity of peptGL^ZIKALIVax^. Consequently, a mutant peptGL^ZIKALIVax^-(T8, I12) was generated and tested with individual anti-ZIKALIVax immune sera (*n* = 7) showing a marked immune reactivity at serum dilution 1:200 (Figure 3A). Peptide-based ELISA showed that the permutation (I8, T12) has no impact on reactivity of anti-ZIKALIVax immune sera with peptGL^ZIKALIVax^ (Figure 3A). Next, we examined whether His residue at peptide position 14 contributes to the lack of peptGL^ZIKALIVax^ recognition by anti-ZIKALIVax immune sera. For this purpose, we generated a new mutant peptGL^ZIKALIVax^-(H14) bearing the amino-acid change Tyr-to-His at peptide position 14 (Figure 1A). In contrast to what was been observed with the other amino-acid changes T8I and I12T, the mutation Y14H caused a lack of reactivity of peptGL^ZIKALIVax^ with anti-ZIKALIVax immune sera (Figure 3B). Thus, a Tyr residue at position 14 greatly influences the antigenic reactivity of peptGL^ZIKALIVax^. Taken together, these results showed that a synthetic 20-mer peptide representing ZIKALIVax GL reacts as antibody epitope. We identified an important role for the polar residue at position E-158 in antigenic reactivity of ZIKV GL region in relation with anti-ZIKALIVax immune sera.

### 2.2. Immunogenicity of PeptGL^ZILALIVax^ in Mice

**Figure 4:**
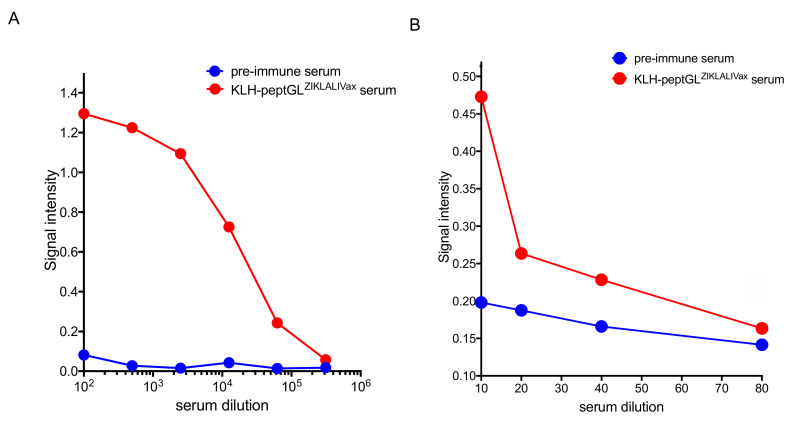
Antigenic reactivity of mouse KLH-peptGL^ZIKALIVax^ immune sera. The O.D_450_ or signal intensity of serum samples through peptide-based ELISA. Pre-immune sera and immune sera from BALB/c mice that received KLH-peptGL^ZIKALIVax^ conjugates were pooled and then tested with KLH-peptGL^ZIKALIVax^ conjugate (**A**) or free peptGL^ZIKALIVax^ (**B**) as coating antigen.

To evaluate the immunogenicity of peptGL^ZIKALIVax^ in inbred laboratory mice, the 20-mer peptide was N-terminally coupled to Keyhole Limpet Hemocyanin (KLH) protein carrier. A group of five adult BALB/c mice were immunized with 20–30 µg of KLH-peptGL^ZIKALIVax^ conjugate in a prime-boost schedule with two weeks lapsing between immunizations. Immune sera were collected three weeks after the third immunization and pooled. Peptide-based ELISA was performed to evaluate the ability of the protein-peptide conjugates to elicit antibody production of relevant specificity (Figure 4). A dose-response curve showed that KLH-peptGL^ZIKALIVax^ conjugate was immunogenic in mice (Figure 4A). Pooled mouse immune sera were then tested on free peptGL^ZIKALIVax^ through a peptide-based ELISA (Figure 4B). A weak antigenic reactivity of anti-KLH-peptGL^ZIKALIVax^ immune sera was detected with peptGL^ZIKALIVax^ at the lower dilution of serum.

**Figure 5:**
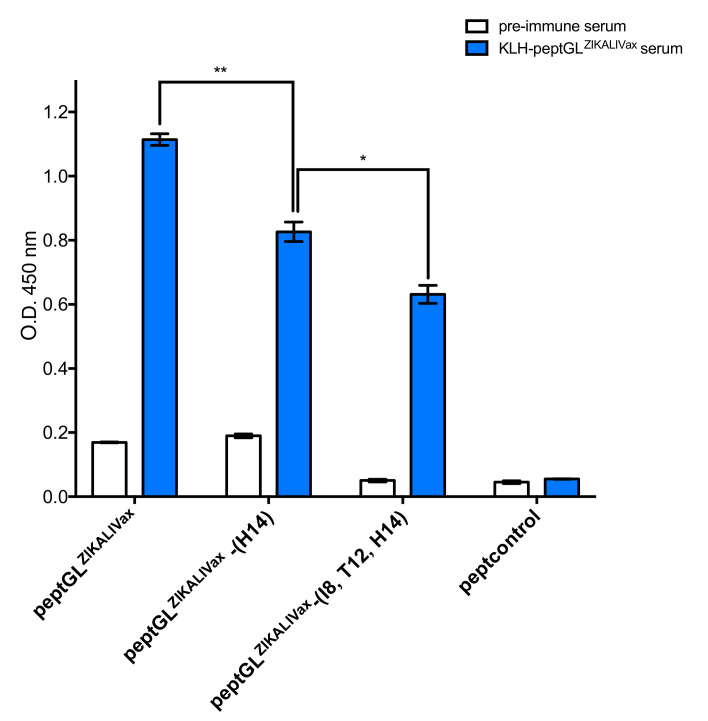
KLH-peptGL^ZIKALIVax^ immune serum reacts with peptGL^ZIKALIVax^. The O.D._450_ or signal intensity of mouse pre-immune and KLH-peptGL^MR766^ immune serum S1-RE (KLH-peptGL^MR766^ serum) at dilution 1:200 in relation to peptGL^ZIKALIVax^ and mutant peptides through peptide-based ELISA. Peptcontrol was used as a negative peptide control. The results are the mean (±S.D.) of three repeats. Statistical values correspond to differences between peptides. Pairwise comparisons were performed and statistically significant comparisons are shown as ** *p* < 10^−3^, * *p* < 10^−2^.

Serum samples of BALB/c mice twice inoculated with KLH-peptGL^ZIKALIVax^ conjugates were tested individually using peptGL^ZIKALIVax^ through peptide-based ELISA. Among the five individual KLH-peptGL^ZIKALIVax^ immune sera tested, only a serum sample from immunized mouse S1-S1-RE (for Series 1-Right Ear) at dilution 1:200 was positive for GL peptide-reactive antibodies (Figure 5). Such a result suggests that peptGL^ZIKALIVax^ is poorly immunogenic in mice. The two mutants, peptGL^ZIKALIVax^-(I8, T12, H14) and peptGL^ZIKALIVax^-(H14), were assayed with KLH-peptGL^ZIKALIVax^ immune serum S1-RE though peptide-based ELISA (Figure 5). The GL peptide-reactive antibodies were capable of reacting with the two peptGL^ZIKALIVax^ mutants but at a lower extent as compared to peptGL^ZIKALIVax^. The amino-acid change T8I decreased GL peptide recognition by KLH-peptGL^ZIKALIVax^ immune serum S1-RE (Figure 5). The GL peptide-reactive antibodies showed a much lower reactivity with mutant peptGL^ZIKALIVax^-(I8, T12, H14) representing BeH819015 GL region. Taken together, these results showed that KLH-peptGL^ZIKALIVax^ immune serum S1-RE has ability to react with GL region of ZIKALIVax. However, the efficiency of antibody binding to ZIKV GL region was weaker with epidemic viral strain BeH819015, which differs from ZIKALIVax by the amino-acid substitutions at positions E-152/156/158.

Using a flow cytometry neutralization test (FNT) (37), we were unable to detect ZIKV neutralizing antibodies in KLH-peptGL^ZIKALIVax^ immune serum S1-RE (Figure S1A). Moreover, there was no synergic effect of GL-peptide reactive antibody with anti-E mAb 4G2, which recognizes an EDII epitope involved in flavivirus neutralization (Figure S1A). The reactivity of KLH-peptGL^ZIKALIVax^ immune serum S1-RE with ZIKV was also tested on Vero cells infected with viral strain MR766-NIID. By immunofluorescence (IF) analysis, no positive signal was observed in ZIKV-infected cells (Figure S1B). Thus, these virological assays did not allow us to demonstrate that GL peptide-reactive antibodies have ability to react with live ZIKV.

### 2.3. Antigenic Reactivity of ZIKALIVax rEDI in Relation with GL Peptide-Reactive Antibody

**Figure 6:**
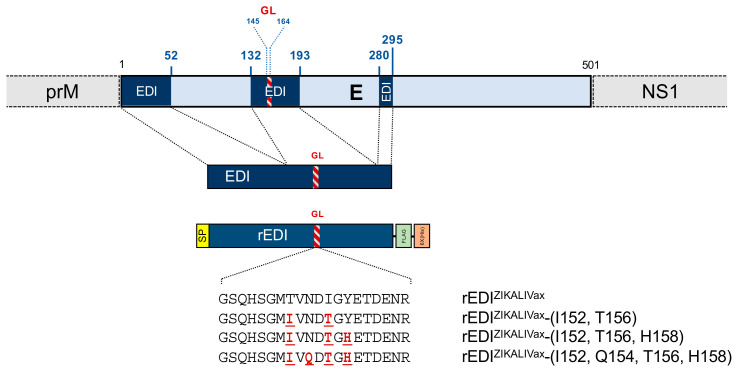
Schematic representation of ZIKALIVax rEDI constructs. The organization of the E protein into ZIKV polyprotein with its three segments composing the EDI domain is shown on top. The EDI residues are numbered as those in the E protein of ZIKALIVax. The glycan loop (GL) sequence of E is shown as a red, hatched segment. The rEDI^ZIKALIVax^ sequence is preceded by a N-terminal heterologous signal peptide (SP) and followed by the spaced two C-terminal FLAG and 6x(His) tags in tandem. The rEDI^ZIKALIVax^ gene was inserted into plasmid vector pcDNA3. Directed mutagenesis was performed on rEDI^ZIKALIVax^ sequence to generate three mutants. The amino-acid substitutions introduced into rEDI^ZIKALIVax^ are indicated in red bold.

The above results prompted us to determine whether KLH-peptGL^ZIKALIVax^ immune serum S1-RE can recognize GL region into a reccombinant EDI domain (rEDI) that regroups 132 discontinuous amino-acid residues of Zika E protein. A recombinant rEDI was, therefore, constructed by joining together the three ZIKV E segments (residues 1 to 52), (residues 132 to 193), and (residues 280 to 294), which compose EDI of ZIKALIVax using a mammalian codon-optimized synthetic gene (Figure 6). The middle EDI segment contains the 20 residues E-145 to E-164 of the GL sequence. The synthetic gene coding for recombinant ZIKALIVax EDI (rEDI^ZIKALIVax^) was inserted into pcDNA3 expression vector to generate a recombinant plasmid pcDNA3/rEDI^ZIKALIVax^. The rEDI^ZIKALIVax^ sequence was preceded by a heterologous signal peptide to target the recombinant viral protein into the secretory pathway and ended with two spaced C-terminal tags, the FLAG and 6 × (His) epitopes in tandem (Figure S2A).

The amino-acid substitutions T152I, I156T, and Y158H were introduced in pcDNA3/rEDI^ZIKALIVax^ by directed mutagenesis to generate a mutant plasmid pcDNA3/rEDI^ZIKALIVax^-(I152, T156, H158) (Figure 6). As a control, we generated a mutant rEDI^ZIKALIVax^ -(T152, Q154, I156, Y158) bearing the amino-acid change Asn to Gln at position E154. It is expected that mutation Q154 causes a loss of the N-glycosylation site of mutant rEDI^ZIKALIVax^-(I152, T156, H158). According to the role of N-glycosylation in antigenic reactivity of Zika E protein [29,30], the amino-acid substitutions T152I and I156T were introduced into rEDI^ZIKALIVax^ leading to a N-glycosylation sequon Asn-Asp-Thr into the rEDI^ZIKALIVax^ GL region (Figure 6).

**Figure 7:**
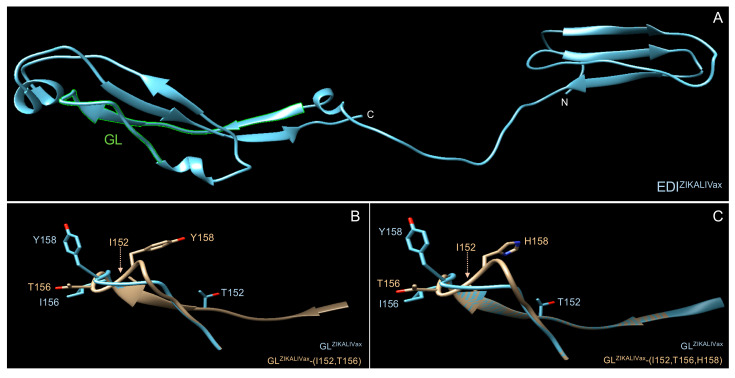
Structural features of rEDI^ZIKALIVax^. Tridimensional structure prediction of rEDI ^ZIKALIVax^ and its mutants was performed by comparative modeling on PHYRE^2^ protein recognition server (http://www.sbg.bio.ic.ac.uk/~phyre2/html/page.cgi?id=index). The predicted structures were analyzed with Chimera, a program for interactive visualization of tridimensional molecules. In (**A**), predicted structure of ZIKALIVax rEDI with GL region in green. The two C-terminal tags of rEDI are not presented. In (**B**,**C**), the ZIKALIVax GL region structure (GL^ZIKALIVax^) was compared to GL mutants bearing the two mutations E-T152I, and E-I156T (**B**) or the three mutations E-T152I, E-I156T, and E-Y158H (**C**). The hatched arrow indicates the position of residue I152 in the 3D structure of GL mutant.

Using BepiPred-2.0 web site (http://www.cbs.dtu.dk/services/BepiPred/) to predict B-cell epitopes located into proteins [38], GL region was identified as a potential antibody epitope in both rEDI^ZIKALIVax^ and rEDI^ZIKALIVax^ -(T152, I156, Y158) mutant, the latter representing the BeH819015 GL sequence (Figure S2B). A 3D structure prediction of ZIKALIVax rEDI was performed using PHYRE^2^ protein fold recognition server (Figure 7). Structural analysis of rEDI showed that the mutations E-T152I and E-I156T may have an effect on GL conformation. The presence of these two mutations might also affect the positioning of residue E-158 into GL region (Figure 7). Thus, the GL region structure might be impacted by the residues E-152 and E-156.

**Figure 8:**
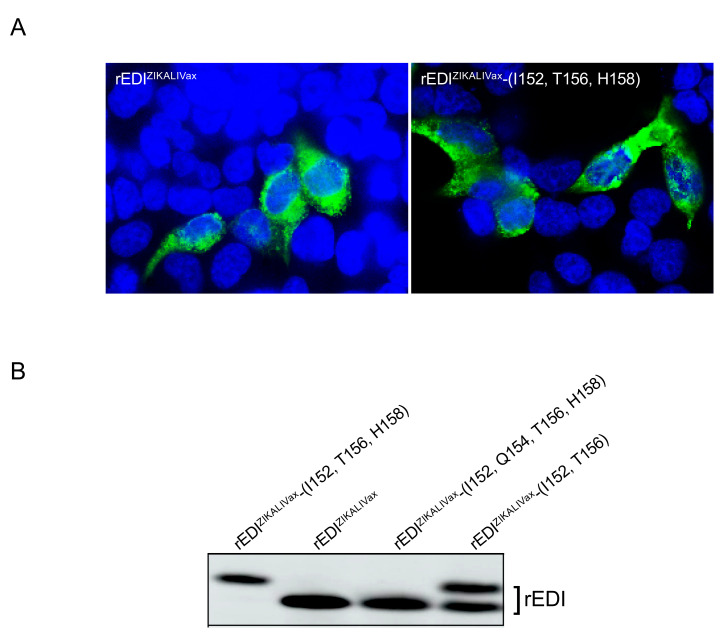
Expression of rEDI^ZIKALIVax^ in HEK-293T cells. HEK-293T cells were transfected 24 h with plasmids pcDNA3 expressing rEDI^ZIKALIVax^ or its mutants. In (**A**), IF assay was performed on fixed and permeabilized cells with anti-6x(His) monoclonal antibody (mAb) as primary antibody. Alexa 488-conjugated anti-mouse IgG antibody as secondary antibody. The nuclei were stained with DAPI (blue). Immunostained cells were visualized with a fluorescent microscope. The same magnification of ×100 was used throughout. In (**B**), immunoblot was performed on RIPA lysates obtained from transfected cells expressing rEDI^ZIKALIVax^ or its mutants using anti-FLAG antibody.

We first evaluated the expression level of rEDI^ZIKALIVax^ and its mutant rEDI^ZIKALIVax^-(I152, T156, H158) by IF assay (Figure 8A). Human epithelial HEK-293T cells were transfected 24 h with plasmids expressing rEDI tagged with 6 × (His) epitope at the C-terminus. IF assay with anti-6 × (His) antibody readily detected intracellular expression of rEDI^ZIKALIVax^ and its mutant rEDI^ZIKALIVax^-(T152, I156, Y158) in transfected HEK-293T cells (Figure 8A). There was no obvious change in the subcellular distribution between the two rEDI. Immunoblot assay on RIPA cell lysates was performed for assessing rEDI protein expression in transfected HEK-293T cells (Figure 8B). Anti-FLAG antibody detected the expression of rEDI^ZIKALIVax^ and its three mutants in HEK-293T cells. We observed that rEDI^ZIKALIVax^ migrated faster than mutant rEDI^ZIKALIVax^-(I152, T156, H158). The fact that rEDI^ZIKALIVax^ and mutant rEDI^ZIKALIVax^-(I152, Q154, T156, H158) have comparable migration profiles is consistent with a lack of glycan linked to rEDI. We can, therefore, consider that the faster migration of rEDI^ZIKALIVax^ is essentially due to the absence of N-glycosylation. In contrast to what was observed with rEDI^ZIKALIVax^-(I152, T156, H158), the rEDI^ZIKALIVax^-(I152, T156) mutant migrated as two closely spaced bands, suggesting that N-linked protein glycosylation of rEDI was not finalized in HEK-293T cells (Figure 8B). Structural protein analysis of glycosylated rEDI^ZIKALIVax^ mutants did not allow us to observe a conformational change explaining the lower occupancy of the N-glycosylation site in rEDI^ZIKALIVax^-(I152, T156) (Figure S3). Thus, it is unlikely that polar residue Tyr at position E-158 of rEDI^ZIKALIVax^-(I152, T156) mutant may have an effect on glycan-protein linkage leading to partially glycosylated rEDI.

**Figure 9:**
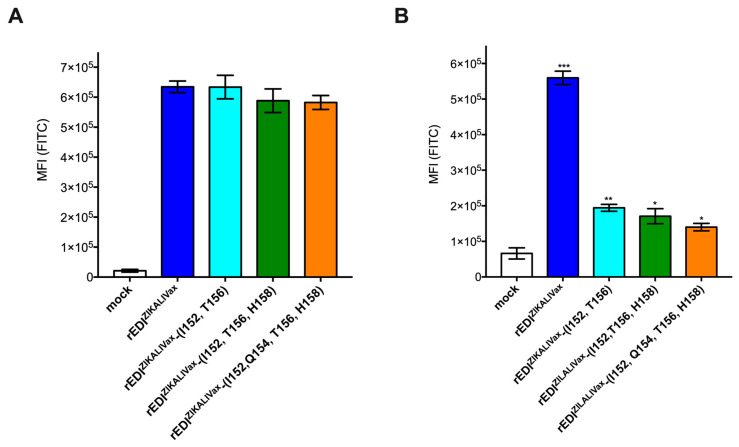
Recognition of rEDI^ZIKALIVax^ in relation with peptGL^ZIKALIVax^-reactive antibody. HEK-293T cells were transfected 24 h with plasmids expressing rEDI^ZIKALIVax^ or its mutants or mock-transfected (mock). FACS analysis was performed to detect expression of rEDI in transfected cells. For FACS analysis, cells were incubated with anti-FLAG antibody (**A**) or KLH-peptGL^MR766^ immune serum S1-S1-RE (KLH-peptGL^MR766^ serum) (**B**) as primary antibody and then Alexa 488-conjugated anti-mouse IgG antibody as secondary antibody. The MFI values of FITC were determined. The error bars represent standard errors of two independent experiments. In (**A**), MFI values generated by the three rEDI^ZIKALIVax^ mutants in comparison with rEDI^ZIKALIVax^. In (**B**), MFI values generated by rEDI^ZIKALIVax^ and its mutants in comparison with mock. Pairwise comparisons were performed and statistically significant comparisons are shown as *** *p* < 10^−2^, ** *p*< 10^−1^, * *p* < 0.05. The nonstatistically significant comparisons are not indicated.

FACS analysis was performed to evaluate the reactivity of KLH-peptGL^ZIKALIVax^ immune serum S1-RE with rEDI^ZIKALIVax^ and its three mutants expressed in HEK-293T cells (Figure 9). Anti-FLAG antibody showed that their expression levels were similar at 24 h post-transfection (Figure 9A). As shown in Figure 9B, KLH-peptGL^ZIKALIVax^ immune serum S1-RE has ability to react with rEDI^ZIKALIVax^ expressed in HEK-293T cells. The mean fluorescence intensity (MFI) values of GL peptide-reactive antibody and anti-FLAG antibody were similar, indicating a high level of rEDI^ZIKALIVax^ antigenic reactivity in relation with GL peptide-reactive antibodies. Introduction of amino-acid substitutions E-T152I, E-I156T, and E-Y158H reduced by at least 70% the antigenic reactivity of rEDI^ZIKALIVax^ (Figure 9B). Analysis of mutant rEDI^ZIKALIVax^-(I152, T156) revealed that the two amino-acid substitutions E-T152I and E-I156T decreased recognition of rEDI^ZIKALIVax^. Given that amino-acid change E-I156T generates a potential sequon Asn-Asp-Thr into GL region of rEDI^ZIKALIVax^ (Figure S2), the amino-acid substitution N154Q was introduced in glycosylated mutant rEDI^ZIKALIVax^-(I152, T156, H158) (Figure 9B). The loss of the N-glycosylation site showed no effect on immunoreactivity of mutant rEDI^ZIKALIVax^. Thus, it seems unlikely that the glycan linked to N154 may play a role in the weaker reactivity of GL peptide-reactive antibody in relation with rEDI^ZIKALIVax^ mutants bearing the mutations E-T152I and E-I156T (Figure 9B). Taken together, these results showed that GL peptide-reactive antibody has ability to recognize the GL region into a reconstituted EDI of ZIKALIVax regardless of the presence of a glycan linked to N154. The two residues E-152 and E-156 might have a key role in the antigenic reactivity of GL region in relation with a change in GL structural conformation.

## 3. Discussion

Recently, we designed the chimeric viral clone ZIKALIVax as a prototype of live-attenuated ZIKV strain for the development of vaccine candidates against Zika disease [36]. ZIKALIVax is a nonglycosylated ZIKV with structural protein region from Asian BeH819015 strain and African MR766-NIID strain as viral backbone [36]. It is of note that MR766 is a laboratory-adapted viral strain since the first isolation in Uganda in 1947. The E protein of ZIKALIVax, which relates to BeH819015 sequence, was modified with the three amino-acid substitutions I152T, T156I, and H158Y, leading to a lack of N-glycosylation site for GL into the E antigenic domain I. The amino-acid residues T152, I156, and Y158 were originally identified in viral strain MR766-NIID. Our in vivo experiments identified a role for the GL region in the reactivity of anti-ZIKALIVax immune serum in relation with ZIKV strains of different lineages [36,37]. Viral strain MR766-NIID as well as ZIKALIVax were highly sensitive to neutralization by the mouse anti-ZIKALIVax immune serum. However, there was a weaker antibody-mediated neutralization of viral strain BeH819015 by anti-ZIKALIVax immune serum, despite the fact that neutralizing antibody epitopes came from BeH819015 [36]. We postulated that glycan loop residues E-152/156/158 may have an impact on availability of neutralizing antibody epitopes on ZIKV surface [36].

The aim of our study was to better understand the role of glycan loop region in the antigenic properties of ZIKALIVax containing the mutations E-I152T, E-T156I, and E-H158Y in the BeH819015 E protein. For such a purpose, we decided to evaluate the antigenic reactivity of a synthetic 20-mer peptide GSQHSGMTVNDIGYETDENR representing the GL region of ZIKALIVax in relation with a mouse anti-ZIKALIVax immune serum. The B-cell epitope prediction model identified the ZIKALIVax GL peptide as a potential antibody epitope. Peptide-based ELISA revealed that a limited number of mice inoculated with ZIKALIVax were capable of reacting with the glycan loop peptide. This would suggest that the glycan loop region is weakly immunogenic in mice challenged with ZIKV.

We noted that a ZIKALIVax GL peptide mutant bearing the amino acid change Tyr to His at position 14 (referred to as residue E-158 in ZIKV) lacks antigenic reactivity in relation with anti-ZIKALIVax immune serum. It was observed that GL-reactive antibodies raised against Asian-lineage ZIKV were inefficient to recognize viral strain MR776 bearing a residue Y158 [34]. The H158 protonation in the GL region might have an effect on the low pH-dependent fusion step at the early stage of virus replication cycle [32]. This suggests an important role for residue E-158 in the structural arrangement of ZIKV. It would be, therefore, of great interest to determine whether a ZIKALIVax mutant bearing a mutation Y158H into the GL region has ability to produce higher levels of neutralizing antibodies against epidemic ZIKV strains of Asian lineage.

To evaluate the ability of ZIKALIVax GL peptide to induce production of GL peptide-reactive antibodies, the peptide conjugated to protein carrier KLH was administrated to a small group of adult BALB/c mice by the intradermal route. Only one individual from five immunized mice developed a significant level of GL peptide-reactive antibodies. The weak immunogenicity of ZIKALIVax GL peptide correlates with the limited number of individual anti-ZIKALIVax immune sera showing antibody reactivity with GL peptide. The mouse immune serum raised against ZIKALIVax GL peptide was capable of reacting with a peptide representing the GL region of viral strain BeH819015. Thus, immunization with ZIKALIVax GL peptide induces the production of GL peptide-reactive antibodies against ZIKV strains of African and Asian lineages. The serum specimen obtained from mouse inoculated with ZIKALIVax GL peptide was assayed with live ZIKV. The GL peptide-reactive antibodies were unable to neutralize ZIKV or even to recognize the authentic E protein. It cannot rule out that sensitivity of our assays was not sufficient to evaluate the binding capacity of GL peptide-reactive antibodies to live ZIKV. In flavivirus E dimers, the hydrophobic fusion-peptide (FP) loop within the E domain II (EDII) nests into a pocket at the interface between EDI and the E domain III [39]. Structural analysis of ZIKV identified GL as a region adjacent to FP loop that is inaccessible in the mature virus structure [18,19,31]. During the low-pH-triggered flavivirus fusion process, the E dimers undergo structural transitions leading to the exposure of the FP loop at the tip of EDII [40]. Although experimental evidence for such a hypothesis is still lacking, we proposed that accessibility of ZIKV GL region for antibody binding requires that ZIKV virion adopts a conformational transition, which results in a change in the oligomeric state of E.

The lack of antigenic reactivity of live ZIKV in relation with GL peptide-reactive antibodies prompted us to determine whether the E-Domain I, which encompasses the GL region, could be recognized by immune serum of mouse that received ZIKALIVax GL peptide. Consequently, a recombinant rEDI was generated by joining the three E segments that compose the 132-amino-acid-long residues of BeH819015 EDI. The introduction of the three amino-acid changes E-I152T, E-T156I, and E-H156Y in BeH819015 EDI resulted in ZIKALIVax EDI. Based on protein structural information from the 3D predicted structure of rEDI, the two amino-acid substitutions E-T152I and E-I156T might have an effect on GL conformation leading to a change in residue E-158 positioning. The serum specimen obtained from mouse inoculated with ZIKALIVax GL peptide reacts with ZIKALIVax rEDI expressed in HEK-293T cells. The impact of residues E-152, E-156, and E-158 on the antigenic reactivity of ZIKALIVax rEDI was assessed through two mutants bearing the amino-acid changes E-T152I, E-I156T, and E-Y158H or only E-I152T and E-I156T. We showed that the mutations E-I152T and E-I156T reduced antigenic reactivity of ZIKALIVax rEDI in relation with GL peptide-reactive antibodies. In order to better understand the role of glycan linked to N154 in the antigenic reactivity of GL region, the amino-acid substitution E-N154Q was introduced into ZIKALIVax rEDI-(I152, T156, H158) mutant. We showed that the weaker antigenic reactivity of ZIKALIVax rEDI mutants bearing the mutation I156T in relation to GL-reactive peptide antibodies did not relate to glycan linked to N154.

In the present study, we demonstrated that a peptide representing the glycan loop region of chimeric viral clone ZIKALIVax reacts as a linear B-cell epitope. This is consistent with the previous reports on the immune reactivity of Zika patient sera with ZIKV GL region [34] and the ability of a recombinant soluble E protein of Asian-lineage ZIKV strain to induce the production of GL-reactive antibodies in mice [35]. Our data reveal that the three residues E-152, E-156, and E-158 have major impact on the antigenic reactivity of ZIKV glycan loop.

## 4. Conclusions

A major role has been proposed for the E glycan loop region (residues E-151 to E-165) located in the domain I of the E protein in the neutralizing activity of anti-ZIKV antibodies [31,32,33,34,35,36]. It has been recently reported that glycan loop of Asian-genotype ZIKV encompasses a highly conserved linear epitope, with His residue at position E-158 playing a key role in the antibody binding [34]. Consistent with this finding, we showed that sera of mice that received live ZIKALIVax, which contains the E protein of contemporary epidemic strain BeH819015 with the residues E-152/156/158 from African strain MR766-NIID, are capable of reacting with a synthetic 20-mer peptide representing ZIKALIVax GL region. Our data demonstrated that polar amino acid at position E-158 greatly influences the antigenic reactivity of glycan loop region between African- and Asian-lineage ZIKV. The residues E-152 and E-156 are proposed to be critical for antigenic reactivity of glycan loop region in relation with the conformational state of E domain I. It is of note that N154-linked glycan has no effect on the binding of ZIKALIVax GL peptide-reactive antibodies to GL region of Asian-genotype ZIKV.

It has been reported that a GL-related peptide conjugated to viral nanoparticles has ability to induce antibodies raised against the glycan loop region of Asian-lineage ZIKV [34]. We demonstrated here that immunization of BALB/c mice with ZIKALIVax GL-peptide conjugated to an immunogenic protein carrier results in production of GL peptide-reactive antibodies capable of reacting with ZIKV strains of different lineages. We and others pointed out the major role of glycan loop region in the antigenic reactivity of ZIKV E protein [31,32,33,34,35,36]. Taken in consideration with the singularity of ZIKV glycan loop region among flavivirus E proteins, the 20-mer peptide GSQHSGMTVNDIGYETDENR representing the ZIKALIVax GL sequence could be of major interest in the design of a peptide-based ELISA for ZIKV infection serological diagnosis [41]. Such a peptide would be also of great interest to provide a clearer view on the involvement of glycan loop region into the E-Domain I on the availability of neutralizing antibody epitopes on ZIKV surface.

## 5. Materials and Methods

### 5.1. Cells and Reagents

Vero (clone E6) and HEK-293T cells were cultured at 37 °C under 5% CO_2_ in Dulbecco’s Modified Eagle Medium (DMEM) growth medium supplemented with 5 to 10% of heat-inactivated fetal bovine serum (Dutscher, Brumath, France) and antibiotics. Mouse anti-flavivirus E mAb 4G2 was purchased from RD-Biotech (Besançon, Fance). Mouse anti-DDDDK tag (binds to FLAG tag sequence) and mouse anti-6 × (His) mAbs were purchased from Abcam (Birmingham, UK). Donkey anti-mouse IgG, HRP conjugate, horseradish peroxidase (HRP) was purchased from ImmunoReagents (Raleigh, USA). Donkey anti-mouse IgG, Alexa Fluor 488 conjugate, was purchased from Thermo Fisher Scientific (Les Ulis, France). DAPI was purchased from Euromedex (Souffelweyersheim, France).

### 5.2. Synthetic Zika Peptides

A synthetic peptide representing the 20 amino-acid residues GSQHSGMTVNDIGYETDNER of the envelope glycan loop of chimeric viral clone ZIKALIVax was chemically synthesized by Genecust (Boynes, France). ZIKALIVax GL peptide mutants and peptcontrol peptide were also produced by Genecust (Boynes, France). The peptides peptGL were provided free or conjugated to the carrier protein keyhole limpet hemocyanin (KLH) by Genecust (Boynes, France).

### 5.3. Mouse Immunization with Peptides

A group of adult BALB/c mice (*n* = 5) were inoculated with 20–30 µg of KLH-peptGL^ZIKALIVax^ conjugates in Complete Freund’s adjuvant (Sigma, France) by intradermal administration. Two weeks after primary immunization, mice were twice boosted with the same antigen in incomplete Freund’s adjuvant with 2–3 weeks lapsing between immunizations. Mice were bled two weeks after the last immunization. Mouse immune sera were pooled or tested individually.

The protocols and subsequent experiments in mice were ethically approved by the French Ministère de l’Enseignement Supérieur, de la Recherche et de l’Innovation with reference APAFIS#23095-2019111814567346 v3 (1 April 2020). All animal procedures were performed in accordance with the European Union legislation for the protection of animals used for scientific purposes (Directive 2010/63/EU). Experiments were conducted following the guidelines of the Office Laboratory of Animal Care at the Cyclotron and Biomedical Research CYROI platform.

### 5.4. Peptide-Based ELISA

A 96-well plate was coated with 0.1 mL of peptide diluted in PBS at 4 °C overnight. Plates were incubated with mouse sera diluted in PBS-Tween supplemented 3% milk at 37 °C. The plates were washed in PBS-Tween and then incubated in the presence of HRP-conjugated anti-mouse IgG antibody. After washes in PBS-Tween, plates were incubated with TMB substrate solution (Thermo Fisher Scientific, les Ulis, France) and absorbance was measured at 450 nm.

### 5.5. Expression of Recombinant ZIKV EDI Proteins

A mammalian codon-optimized gene coding for a heterologous signal peptide, followed by the amino-acid residues (1–52), (132–193), and (280–295) that compose the EDI domain of chimeric viral clone ZIKALIVax and ended by FLAG and 6 × (His) tags in tandem with glycine-serine spacers, was synthetized by Genecust (Boynes, France) (Figure S2A). The synthetic EDI gene was cloned into *Nhe* I and *Not* I restriction site of the pcDNA3.1-Hygro plasmid to generate pcDNA3/ZIKV-rEDI^ZIKALIVax^. The different mutants of rEDI^ZIKALIVax^ were obtained by direct mutagenesis on pcDNA3/ZIKV-rEDI^ZIKALIVax^ and all plasmids were sequence verified by Sanger method (GeneCust, Boynes, France). HEK-293T cells were transiently transfected with pcDNA3/ZIKV-rEDI^ZIKALIVax^ and mutants using Lipofectamine 3000 (Thermo Fisher Scientific, les Ulis, France).

### 5.6. Immunoblot Assay

Cells were lysed with RIPA lysis buffer (Sigma, Lyon, France) containing protease inhibitors. Equal quantity of proteins was loaded on a 15% SDS-PAGE and transferred to nitrocellulose membrane. After blocking of the membrane in PBS supplemented with FBS and Tween-20, the blot was incubated with appropriate dilutions of anti-FLAG antibody as the primary antibody and then HRP-conjugated anti-mouse IgG antibody as secondary antibody. The membrane was exposed on an Amersham imager 600 (GE Healthcare).

### 5.7. Immunofluorescence Assay

For IF analysis, cell monolayers grown on coverslips were fixed with 3.2% paraformaldehyde in PBS and then permeabilized with 0.1% Triton X-100 in PBS. Alternatively, cells were fixed using cold methanol:acetone for 30 min. Cells were incubated with appropriate dilutions of primary mouse antibody in PBS containing 1% bovine serum albumin and then with Alexa 488-conjugated anti-mouse IgG antibody as secondary antibody in the same buffer. Nuclei were stained with DAPI. Vectashield reagent (Cinisciences, Nanterre, France) was used for mounting of the glass coverslips. A Nikon Eclipse E2000-U microscope was used to visualize the fluorescence. Fluorescent signal capture was allowed with a Hamamatsu ORCA-ER camera coupled to the imaging software NIS-Element AR (Nikon, Champigny-sur-Marne, France).

### 5.8. Flow Cytometry Analysis

For flow cytometry assay, cells were seeded in 24-well plates and then transfected with pcDNA3/ZIKV-rEDI^ZIKALIVax^ or mutants. After 18 h of transfection, cells were gently harvested by trypsinization, fixed with 3.7% FA in PBS, and then permeabilized with 0.15% Triton X-100 in PBS for 5 min. Samples were incubated with appropriate dilutions of mouse immune serum as primary antibody and then with Alexa 488-conjugated anti-mouse IgG antibody as secondary antibody. Cells were analyzed with a Cytoflex flow cytometer (Beckman Coulter, Villepinte, France).

### 5.9. Statistical Analysis

An unpaired *t* test was used to compare quantitative data. GraphPad Prism was used for all statistical analysis.

## Supplementary Materials

The following are available online at https://www.mdpi.com/1999-4915/12/11/1258/s1. Figure S1. Reactivity of mouse KLH-peptGLZIKALIVax immune serum with ZIKV E protein. Figure S2. Prediction of rEDI antibody epitopes in ZIKALIVax. Figure S3. Location of residue E-158 on the 3D structure of glycosylated rEDI.
