# Supplementary material for: Immune Reactivity of a 20-mer Peptide Representing the Zika E Glycan Loop Involves the Antigenic Determinants E-152/156/158
Source: Viruses. 2020 Nov 5;12(11):1258. doi: 10.3390/v12111258 (PMC7694461; doi:10.3390/v12111258)
Supplement: Supplementary file 1 [file viruses-12-01258-s001.pdf]

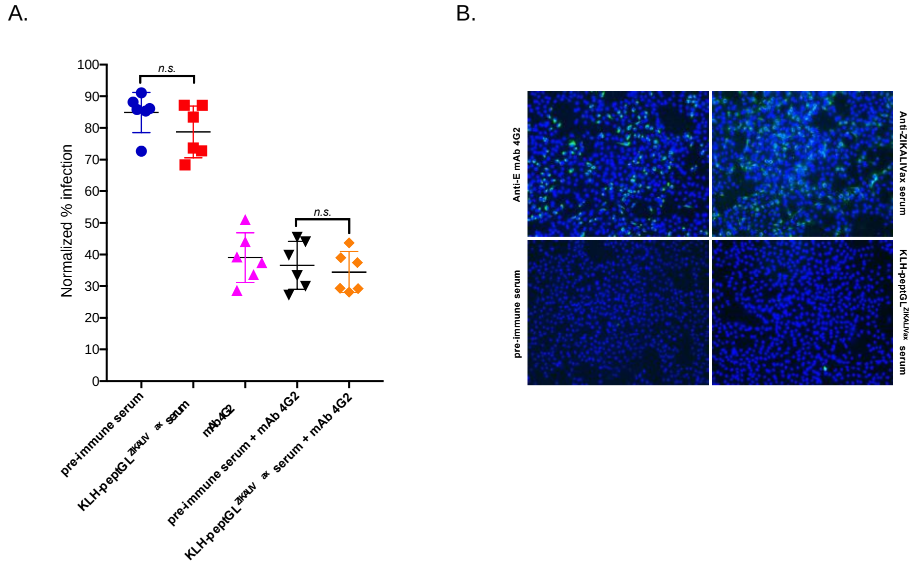

Figure S1. Reactivity of mouse KLH-peptGL^{ZIKALIVax} immune serum with ZIKV E protein. In (**A**), flow cytometry neutralization (FNT) assay on mutant ZIKV^{GFP} was performed. ZIKV^{GFP} sample was mixed with an equal volume of pre-immune or anti-peptGL^{ZIKALIVax} immune serum RE at dilutions of 1:200 or purified anti-E mAb 4G2 or a mix of mAb 4G2 and mouse immune serum. The percentage of GFP-positive cells was determined by flow cytometry analysis. The results were expressed as the percentage of GFP-positive cells in assay relative to that calculated in absence of serum; n.s.: $p > 0.05$. In (**B**), Vero cells were infected 24 h with ZIKV strain MR766 at multiplicity of infection of 1 and then fixed with a mix of methanol/acetone at cold temperature. Mouse immune serum (dilution 1:200) and anti-E mAb 4G2 were used as primary antibody and Alexa 488-conjugated anti-mouse IgG antibody as secondary antibody. The nucleus was stained with DAPI (blue). Immunostained cells were visualized with a fluorescent microscope. The same magnification of x 20 was used throughout.

A.

```
MVLLLILSVLLLKEDVRGSAQSTGLIRCIGVSNRDFVEGMSGGTWVDVVLEHGGCVTVMAQD   rEDI^ZIKALIVax
--------------------------------------------------------------   rrEDI^ZIKALIVax-(I152, T156, H158)

KPTVDIELVTTTVSNPENLEYRIMLSVHGSQHSGMTVNDIGYETDENRAKVEITPNSPRAEA   rEDI^ZIKALIVax
-----------------------------------I---T-H--------------------   rEDI^ZIKALIVax-(I152, T156, H158)

TLGGFGSLGLDCEPRAKGRLSSGHLKCRLKMgggsgggdykddddkgggsggghhhhhh      rEDI^ZIKALIVax
-----------------------------------------------------------      rEDI^ZIKALIVax-(I152, T156, H158)
```

B.

Predicted peptides:

No.	Start	End	Peptide	Length
1	21	28	QSTGLIRC	8
2	36	38	FVE	3
3	75	80	VSNPEN	6
4	91	110	GSQHSGMIVNDTGHETDENR	20
5	119	130	SPRAEATLGGFG	12
6	138	149	PRAKGRLSSGHL	12
7	155	181	MGGGSGGGDYKDDDDKGGGSGGGHHHH	27

rEDI^{ZIKALIVax}

Predicted peptides:

No.	Start	End	Peptide	Length
1	24	28	GLIRC	5
2	36	37	FV	2
3	73	80	TTVSNPEN	8
4	90	111	HGSQHSGMTVNDIGYETDENRA	22
5	118	131	NSPRAEATLGGFGS	14
6	138	146	PRAKGRLSS	9
7	155	181	MGGGSGGGDYKDDDDKGGGSGGGHHHH	27

rEDI^{ZIKALIVax}-(I152, T156, H158)

(Bepipred Linear Epitope Prediction 2.0)

Figure S2. Prediction of rEDI antibody epitopes in ZIKALIVax. In (**A**), alignment of rEDI^{ZIKALIVax} and its mutant rEDI-(T152, I156, Y158) of 181 amino acids. The glycan loop region is underlined. The mutant rEDI-(T152, I156, Y158) corresponds to the authentic sequence of ZIKV strain BeH819015. The signal peptide is indicated in italic. The two C-terminal FLAG and 6 × (His) tags and spacers are indicated in small letters. In (**B**), list of predicted linear B-cell epitopes based on antibody epitope predicator Bepipred Linear Epitope Prediction 2.0 (IEDB Analysis Resource, www. http://tools.iedb.org/bcell/). The glycan loop region was identified as predicted peptide n°4. The two tag epitopes in tandem at the C-terminus of rEDI were identified as predicted peptide n°7.

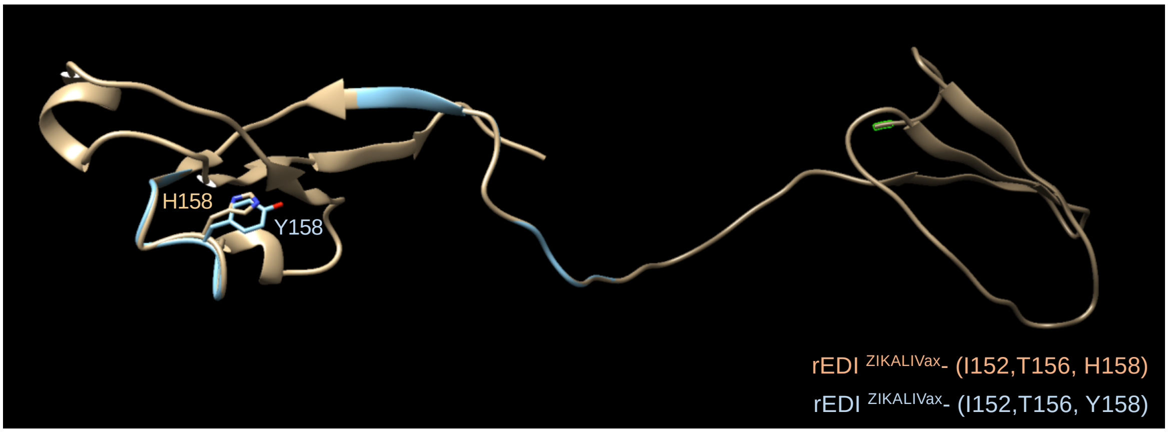

Figure S3. Location of residue E-158 on the 3D structure of glycosylated rEDI. Tridimensional structure prediction of ZIKALIVax EDI sequences bearing the residues I152, T156, and H158 or Y158 by comparative modeling on PHYRE[2] protein recognition server. The predicted structures were analyzed with Chimera.
